# Hand Hygiene Compliance Study at a Large Central Hospital in Vietnam

**DOI:** 10.3390/ijerph16040607

**Published:** 2019-02-19

**Authors:** Cam Dung Le, Erik B. Lehman, Thanh Huy Nguyen, Timothy J. Craig

**Affiliations:** 1 Pennsylvania State College of Medicine, Hershey, PA 17033, USA; camdungle@pennstatehealth.psu.edu (C.D.L.); Elehman@phs.psu.edu (E.B.L.); 2 Department of Public Health Sciences, Pennsylvania State University, Hershey, PA 17033, USA; 3 Department of Infection Control, Hue Central Hospital, Thua Thien Hue 70000, Vietnam; nthuyicdhch@yahoo.com; 4 Division of Allergy, Asthma and Immunology, Penn State Milton S. Hershey Medical Center, Hershey, PA 17033, USA

**Keywords:** hand washing, quality improvement, Vietnam, quality assessment

## Abstract

Lack of proper hand hygiene among healthcare workers has been identified as a core facilitator of hospital-acquired infections. Although the concept of hand hygiene quality assurance was introduced to Vietnam relatively recently, it has now become a national focus in an effort to improve the quality of care. Nonetheless, barriers such as resources, lack of education, and cultural norms may be limiting factors for this concept to be properly practiced. Our study aimed to assess the knowledge and attitude of healthcare workers toward hand hygiene and to identify barriers to compliance, as per the World Health Organization’s guidelines, through surveys at a large medical center in Vietnam. In addition, we aimed to evaluate the compliance rate across different hospital departments and the roles of healthcare workers through direct observation. Results showed that, in general, healthcare workers had good knowledge of hand hygiene guidelines, but not all believed in receiving reminders from patients. The barriers to compliance were identified as: limited resources, patient overcrowding, shortage of staff, allergic reactions to hand sanitizers, and lack of awareness. The overall compliance was 31%; physicians had the lowest rate of compliance at 15%, while nurses had the highest rate at 39%; internal medicine had the lowest rate at 16%, while the intensive care unit had the highest rate at 40%. In summary, it appears that addressing cultural attitudes in addition to enforcing repetitive quality assurance and assessment programs are needed to ensure adherence to safe hand washing.

## 1. Introduction

Hospital-acquired infections (HAIs) are regarded as the most frequent threat to patient safety globally [1]. Not only do HAIs potentially complicate patient treatment and lead to life-threatening conditions, but they can also burden patients economically with prolonged hospital stays [2]. Lack of hand hygiene has been identified as the core facilitator of HAIs. Studies show that healthcare workers only perform hand hygiene less than half as often as they should [3]. Many studies have been conducted to investigate the factors contributing to hand hygiene compliance and indicate that lack of compliance is a concern in hospital settings.

Limited data are collected from developing countries, where resources may be sparse and other emerging health problems and diseases take priority over a surveillance system for HAIs [1]. Hand hygiene compliance education in healthcare settings is a new concept in Vietnam, and the country only recently pledged commitment to the World Health Organization’s (WHO) First Global Patient Safety Challenge—“Clean Care is Safer Care”—in 2009 [4]. Thus, further studies in developing countries such as Vietnam can help to monitor the reception of hand hygiene and to elucidate other factors that hinder hand hygiene practice, such as socioeconomic or cultural norms. In this study, we aimed to assess healthcare workers’ compliance, knowledge, awareness, and attitude regarding hand hygiene, as well as barriers to compliance. For this purpose, we designed a 13-item survey and conducted direct observation to collect data at a large medical center in Vietnam that recently received hand hygiene education from the WHO [4].

## 2. Methods

A total of 11 inpatient service departments were chosen for the study: Five internal medicine services, five pediatrics services, and one intensive care unit service. The five internal medicine services included: hepato-gastroenterology, endocrinology and neurology, nephrology and rheumatology, cardiovascular, and general medicine and geriatrics. The five pediatrics services included: general pediatrics I, general pediatrics II, cardiovascular, pediatric intensive care unit, and neonatal. The subjects of the study were healthcare workers of different positions: physicians, nurses, care assistants, and student nurses.

In order to understand how the limitation of resources, knowledge, and cultural norms affect hand hygiene practice among healthcare workers, we utilized a survey to collect these data. We designed a 13-item survey with questions pertaining to hand hygiene habits, knowledge, awareness, attitude, and resources (Appendix A). To identify preventable factors, the survey investigated systematic factors that might hinder compliance, such as access to utilities, time restrictions due to workload, or lack of training. This survey was translated into Vietnamese. Surveys were given to the head nurse of each service, who distributed the surveys to the rest of the healthcare workers within the service. Each survey was assigned a code to allow the participant to remain anonymous. Data collected from the surveys were entered into a RedCap database for security and confidentiality.

For the direct observation component of the study, we observed and recorded hand hygiene compliance among healthcare workers using the iScrub Lite mobile application [5]. Healthcare workers in each department were randomly observed as they carried out routine care with patients at variable times of the day. Hand hygiene compliance was considered to be performed correctly if the healthcare provider either washed or applied hand sanitizer to his or her hands before and after contact with each patient. Hand washing was the preferred method if the hands were soiled; however, since we did not directly inspect hands, we accepted either method as effective. Observation was conducted for four weeks in all 11 departments during the morning and afternoon shifts when most of the interactions between patients and healthcare workers occurred, and it was conducted in a discreet manner to avoid affecting subjects’ hand hygiene behavior. This was only performed by one observer. Therefore, not every department was observed on each day due to time constraints. On average, about 50 observations were made each day. Observations were recorded on the iScrub Lite application and then exported into an Excel sheet. The data were then compiled to compare compliance across different service departments and different healthcare worker positions. Statistics are description-based only. The project met the Institutional Review Board (IRB) exemption by keeping the anonymity of the participants and by not collecting personal identifiers for both the survey and compliance observation. The study was conducted in July of 2016.

## 3. Results

### 3.1. Demographics

A total of 371 surveys were collected from healthcare workers. Of these, 64 participants were male, 247 were female, and 60 were left unidentified. The number of each healthcare position was identified as follows: 59 physicians, 185 nurses, 27 care assistants, 71 student nurses, and 29 unidentified. The majority of nurses were female. The distribution of surveys across the inpatient service departments was collected as follows: 30 from hepato-gastroenterology, 35 from endocrinology and neurology, 23 from nephrology and rheumatology, 55 from cardiovascular (of the internal medicine department), 29 from general medicine and geriatric; 31 from general pediatrics I, 30 from general pediatrics II, 10 from pediatric cardiovascular, 10 from pediatric intensive care, and 22 from neonatal.

### 3.2. Survey

Across all positions, healthcare workers demonstrated a knowledgeable response regarding the timing of hand hygiene performance and its association with hospital-acquired infections, which is consistent with the WHO’s education (Table 1). There was a mix of attitudes toward patient involvement in reminding healthcare workers to perform hand hygiene. The majority of healthcare workers, more than 86.2%, claimed that their hospital provided convenient utilities to perform hand hygiene. However, when asked to rate how convenient it was to perform hand hygiene before and after each patient contact, most rated “convenient” over “very convenient”. Physicians had the lowest rate (67.2%) of self-reported hand hygiene compliance, while nurses had the highest rate (97.8%), followed by care assistants (96.1%) and student nurses (88.4%). Regarding the timing of hand hygiene performance, most chose “before and after each patient contact”, followed by the “start and end of a shift”. The response was split almost equally between “yes” and “no” regarding whether they were ever reminded by another healthcare worker or a patient to perform hand hygiene. This is in exception to care assistants, where 33.3% said “yes” and 66.7% said “no”. While most healthcare workers believed that their colleagues adhered to the “5 Moments for Hand Hygiene” instructions by the WHO, only 55.9% of the physicians believed this to be true and 37.3% were unsure. From 82.8% to 92.6% of physicians, nurses, care assistants, and student nurses claimed that they would remind their colleagues to follow the hand hygiene guidelines. However, only 37.0% to 57.6% of physicians, nurses, care assistants, and student nurses felt that patients should be involved in reminding healthcare workers to perform hand hygiene. Upon rating how relevant they thought hand hygiene was to preventing the spread of infection effectively in the hospital, about 80% of all positions rated “very relevant”, except for care assistants (60%).

For the open response question (number 11) regarding what barriers within the hospital made it difficult for healthcare workers to adhere to the hand hygiene guidelines, there were several common responses that were seen (Table 2). These included a lack of hand hygiene supplies, inconvenient placement of the supplies, patient overcrowding, work overload and pressure, skin reactions to hand sanitizers, lack of awareness, old habits of not washing hands, and forgetfulness. When asked about ways their hospital could make it easier for healthcare workers to perform hand hygiene, healthcare workers provided open responses that commonly appeared across the surveys. These included providing more supplies, placing the supplies at more convenient locations, decreasing workload, providing hand sanitizer that is more suitable for the skin, reinforcing a punishment and reward policy, and giving frequent verbal reminders.

### 3.3. Observation

A total of 997 observations were made. These included 261 physicians, 327 nurses, 25 care assistants, and 384 student nurses (Table 3). Physicians had the lowest rate of compliance at 14.6%, while nurses had the highest rate of compliance at 38.8%. When compared among departments, the intensive care unit had the highest rate of compliance at 40.5%, followed by pediatrics at 34.5%, and internal medicine at 16.4% (Table 4). Within pediatrics and internal medicine, the cardiovascular service had the lowest compliance at 8.3% and 9.1%, respectively. Overall, the total compliance was 30.6%.

## 4. Discussion

Studies that investigate factors leading to poor hand hygiene compliance among healthcare workers have shown both intrinsic and extrinsic factors. These include forgetfulness, fear of skin damage, lack of time, inadequate supply, inconvenient accessibility, and lack of knowledge and training to educate staff on how and when to practice hand hygiene during their routine [3]. In addition, other important factors include social influence, attitude, and intention [2]. These published findings are consistent with what we found from our survey, specifically from the open response question (number 12) where healthcare workers were asked to identify barriers. At the same time, however, the majority of healthcare workers claimed that, at the beginning of the survey, their hospital provided convenient utilities and locations for them to perform hand hygiene and that they and their colleagues followed hand hygiene guidelines. This inconsistency between claiming the ability to perform hand hygiene and the many barriers that hinder this ability was elucidated when talking to some of the hospital employees, which was not part of the study. One of the theoretical explanations is that healthcare workers do not feel comfortable giving honest answers to a survey regarding themselves and their hospital. It is a common practice in Vietnamese culture that people want to avoid losing face, where face is both an individual and a collective quality [6]. Consequently, even in an anonymous survey, they might not want to report negative views about their hospital. Thus, when faced with “yes” or “no” questions where the participant could easily perceive which choice would put him or herself in a better light, the choice would be swayed. However, when given open response questions, participants provided more valid, elaborative responses.

One interesting factor that we identified as a possible contributor to poor hand hygiene among healthcare workers in Vietnam is the lack of patient involvement. From the survey, we discovered that, while more than 82.8% across all healthcare positions claimed that they would remind their colleagues to perform hand hygiene, most of them did not think that patients should be involved in reminding healthcare workers. Only 57.6% of physicians, 49.7% of nurses, 37% of care assistants, and 56.7% of student nurses felt that patients should be involved in reminding healthcare workers. This showed that, in Vietnam, patients are not encouraged to challenge their healthcare providers. As noted, Vietnam’s emphasis on hierarchic authority causes patients to adopt a passive role in their care [7]. Thus, it would be seen as inappropriate for patients to remind physicians or other healthcare workers to perform hand hygiene. This is unlike American culture, where patient-centered care is widely emphasized and patients are educated to question their doctor. In addition, recent US hand washing campaigns empower patients to ask their doctor if they have washed their hands, such as the Centers for Disease Control and Prevention’s (CDC) Clean Hands Count campaign.

The lack of patient involvement also implies that hand hygiene is not a communal responsibility. This is consistent with another study that was also conducted at the same institution [4]. In addition to addressing barriers such as accessibility to sinks, overcrowded wards, and lack of clean and continuous water, this study also indicated that an important factor was the lack of community hand hygiene practice [4]. In a different study [8] conducted at six hospitals in Hanoi, Vietnam, none of the healthcare workers in the study acknowledged hand hygiene as their “duty of care” toward their patients, since visitors did not have to perform hand hygiene. The study did show, however, that healthcare workers did acknowledge hand hygiene as a duty of care toward themselves. Again, this shows that Vietnam, with regard to hand hygiene practice, is still struggling to view this as a communal responsibility in the hospital setting. This lack of communal practice likely causes healthcare workers to feel less responsible to perform hand hygiene on a routine basis.

While healthcare workers demonstrated a knowledgeable response and claimed compliance with hand hygiene practice on the survey, our direct observation suggested otherwise. Across the board, compliance was only 30.6%. This is similar to what was reported by the WHO, which looked at hand hygiene compliance among healthcare workers in studies conducted from 1981 to 2008. The compliance rate from these studies ranged from 5% to 89%, with an overall average of 38.7% [9]. Our result is also consistent with other studies in other countries, where physicians have sub-optimal compliance rates compared to other healthcare workers [10,11,12]. Our study showed that the physician compliance rate was only 14.6%, while that of nurses was 38.8%. In a much bigger study by Pigget et al. [11], the physician rate was 30%, while that of nurses was 52%.

Even though physicians claimed the same barriers to hand hygiene as other healthcare workers in the surveys, it is possible that there are other intrinsic factors that cause physicians to consistently have lower compliance rates across nations. It is possible that having seniority in title or clinical experience allows physicians to feel more confident with their behavior and judgment regarding when to perform hand hygiene. In a focus group study that looked at factors specific to physicians, overconfidence regarding personal judgment and skepticism toward the hand hygiene guidelines were found to be significant factors in the noncompliance of physicians with respect to hand hygiene practice [13]. Another factor that the study identified was the role of the medical hierarchy, where the junior physician’s hand hygiene behavior is affected by the role modeling of a senior physician in the team. This shows that personal hand hygiene practice is significantly affected by one’s observation of other people’s attitude toward it. Thus, compliance rates could rely heavily on the practice of the whole community.

There are several limitations to this study. First, we were unable to receive all the surveys distributed to each department. A number of healthcare workers were either absent, away, or did not want to participate in the survey. Most of these were physicians, and therefore our data for this subgroup were fewer than expected. Second, we received many surveys that came back with the same handwriting or responses that were verbatim from neighboring surveys. This implied that one person was filling out the survey for the others, and that answers were not individual responses, but rather a group discussion. This behavior could have been influenced by cultural factors, where the importance of data authenticity for the survey was not strongly recognized. Efforts have been made to eliminate these data (less than 5%) from the database, but it is possible that not all were identified and removed. Third, “yes” and “no” answers on the survey may have been influenced by the perception of the subjects on what would represent them and/or their hospital better. Having more open responses may have reduced this concern. Another limitation to our study was that we were unable to determine the denominator of healthcare workers missing for the direct observation study, and therefore we were unable to determine the percentage of the total healthcare workers being observed.

## 5. Conclusions

Hand hygiene practice should be continually emphasized and monitored in developing countries as well as in developed countries. Aside from limitations such as resources, workload, and ward overcrowding, cultural norms and individual attitudes also play important roles in hand hygiene compliance. Our study identifies these last two factors as critical in Vietnam. Aside from providing more funding for resources and changing the workload structure, more education campaigns should focus on changing the attitude and behavior of hand hygiene to normalize it as a standard practice in the community, both inside and outside the hospital setting. Lastly, periodically repeating quality assurance programs is key to ensuring adherence. Hand hygiene should be an ongoing process to ensure maximal adherence.

## Figures and Tables

**a:** 

		**Does Your Hospital Provide Convenient Utilities to Perform Hand Hygiene at the Patient Care Area?**		**How Convenient Is It for You to Perform Hand Hygiene before and after Each Patient Contact? (1 = Completely Not Convenient, 2 = Convenient, 3 = Very Convenient)**			**Do You Wash Your Hands between Each Patient Contact?**			**When Do You Perform Hand Hygiene during the Day? (Choice = 1. Start and End of a Shift)**		**When Do You Perform Hand Hygiene during the Day? (Choice = 4. Before and after Each Patient Contact)**	
**Occupation**	**Total**	**Yes (%)**	**No (%)**	**1 (%)**	**2 (%)**	**3 (%)**	**Yes (%)**	**No (%)**	**Sometimes (%)**	**Yes (%)**	**No (%)**	**Yes (%)**	**No (%)**
**Physician**	59	86.2	13.8	25.4	57.6	17.0	67.2	3.5	29.3	59.3	40.7	91.5	8.5
**Nurse**	185	91.3	8.7	18.9	51.4	29.7	97.8	0	2.2	50.8	49.2	97.8	2.2
**Care Assistant**	27	100	0	0	55.6	44.4	96.1	0	3.9	55.6	44.4	85.2	14.8
**Student Nurse**	71	100	0	0	33.8	66.2	88.4	4.4	7.2	25.4	74.6	94.4	5.6

**b:** 

**Have You Ever Been Reminded by Either Another Healthcare Worker or a Patient about Performing Hand Hygiene before and after Patient Contact?**		**Do You Think Your Colleagues Adhere to the “5 Moments For Hand Hygiene” Guidelines Instructed by the World Health Organization?**			**Do You Remind Your Colleagues to Follow the “5 Moments For Hand Hygiene” Guidelines?**		**Do You Think Patients Should Be Involved in Reminding Healthcare Workers to Perform Hand Hygiene?**			**How Relevant Do You Think Hand Hygiene Practice is at Preventing the Spread of Infection Effectively in the Hospital? (1 = Not Relevant, 2 = Relevant, 3 = Very Relevant)**		
**Yes (%)**	**No (%)**	**Yes (%)**	**No (%)**	**Not sure (%)**	**Yes (%)**	**No (%)**	**Yes (%)**	**No (%)**	**Not sure (%)**	**1 (%)**	**2 (%)**	**3 (%)**
53.4	46.6	55.9	6.8	37.3	82.8	17.2	57.6	30.5	11.9	3.4	23.7	72.9
45.4	54.6	85.7	0.6	13.7	82.9	17.1	49.7	40.6	9.7	2.2	19.6	78.2
33.3	66.7	96.3	0	3.7	92.6	7.4	37	48.2	14.8	0	40	60
52.2	47.8	91	1.5	7.5	92.5	7.5	56.7	35.8	7.5	0	17.9	82.1

**Table 2:** Factors affecting hand hygiene compliance.

Are There Barriers within Your Hospital Making It Difficult for Healthcare Workers to Adhere to Hand Hygiene Performance? If So, What Are They?	In What Ways Could Your Hospital Make It Easier for Healthcare Workers to Perform Hand Hygiene?
Not enough hand hygiene supplies such as hand sanitizers, hand sinks, and towels	Provide more hand hygiene supplies
Inconvenient placement of hand hygiene supplies	Place supplies at more convenient locations
Patient overcrowding	Hire more staff or decrease number of patients
Work overload and pressure	Decrease amount of workload
Skin reaction to hand sanitizers	Provide hand sanitizers suitable for skin
Lack of awareness	Reinforce punishment and reward
Old habit of not washing hands/forgetfulness	Give frequent verbal reminders

**Table 3:** Hand hygiene compliance of healthcare workers from direct observation.

Occupation	Number of Observations	Compliant	Non-Compliant
Physician	261	38	223
		14.6%	85.4%
Nurse	327	127	200
		38.8%	61.2%
Care Assistant	25	4	21
		16%	84%
Student Nurse	384	137	247
		35.7%	64.3%

**Table 4:** Hand hygiene compliance of different departments from direct observation.

Department	Number of Observations	Compliant	Non-Compliant
**Internal Medicine**			
Endocrinology and Neurology	68	17 (25%)	51 (75%)
Nephrology and Rheumatology	70	10 (14.3%)	60 (85.7%)
Hepato-Gastroenterology	92	17 (18.5%)	75 (81.5%)
General Medicine and Geriatric	44	5 (11.4%)	39 (88.6%)
Cardiovascular	55	5 (9.1%)	50 (90.9%)
Internal Medicine Total	329	54 (16.4%)	275 (83.6%)
**Pediatrics**			
Neonatal	55	26 (47.3%)	29 (52.7%)
Intensive Care	39	13 (33.3%)	26 (66.7%)
Cardiovascular	12	1 (8.3%)	11 (91.7%)
General Pediatrics I	96	14 (14.6%)	82 (85.4%)
General Pediatrics II	120	57 (47.5%)	63 (52.5%)
Pediatrics Total	322	111 (34.5%)	211 (65.5%)
Intensive Care Unit	346	140 (40.5%)	206 (59.5%)
Total	997	305 (30.6%)	692 (69.4%)

## Appendix A

Healthcare Worker Survey

Survey code: …………………….

Date:………………

Age:………………..

Gender: Male………Female………

Occupation: …………………………….

1. Does your hospital provide convenient utilities to perform hand hygiene at the patient care area? Please circle your answer.

Yes No

2. How convenient is it for you to perform hand hygiene before and after each patient contact? (1 = completely not convenient, 2 = convenient, 3 = very convenient) Please circle your answer.

1 2 3

3. Do you wash your hands between each patient contact? Please circle your answer.

Yes No Sometimes

4. When do you perform hand hygiene during the day? Please circle your answer (circle as many as apply).

1. Start and end of a shift

2. Every hour

3. Before or after each meal

4. Before and after each patient contact

5. Other answer:______________________________

5. How frequently are healthcare workers reminded to perform hand hygiene in your department? Please circle your answer.

1. Every day 2. Weekly 3. Monthly 4. Yearly 5. Never

6. Have you ever been reminded by either another healthcare worker or a patient about performing hand hygiene before and after patient contact? Please circle your answer.

Yes No

7. Do you think your colleagues adhere to the “5 Moments For Hand Hygiene” guidelines instructed by the World Health Organization? Please circle your answer.

Yes No Not sure

8. If you answered “No” to question 7, please provide a reason as to why you think this happens.

9. Do you remind your colleagues to follow the “5 Moments For Hand Hygiene” guidelines? Please circle your answer.

Yes No

10. Do you think patients should be involved in reminding healthcare workers to perform hand hygiene? Please circle your answer.

Yes No Not sure

11. How relevant do you think hand hygiene practice is at preventing the spread of infection effectively in the hospital? Use a scale of 1–3, where 1 = not relevant, 2 = relevant, 3 = very relevant. Please circle your answer.

1 2 3

12. Are there barriers within your hospital making it difficult for healthcare workers to adhere to hand hygiene performance? If so, what are they?

13. In what ways could your hospital make it easier for healthcare workers to perform hand hygiene?
